# IGWO-IVNet3: DL-Based Automatic Diagnosis of Lung Nodules Using an Improved Gray Wolf Optimization and InceptionNet-V3

**DOI:** 10.3390/s22249603

**Published:** 2022-12-07

**Authors:** Anas Bilal, Muhammad Shafiq, Fang Fang, Muhammad Waqar, Inam Ullah, Yazeed Yasin Ghadi, Haixia Long, Rao Zeng

**Affiliations:** 1 College of Information Science and Technology, Hainan Normal University, Haikou 571158, China; 2 School of Information Engineering, Qujing Normal University, Qujing 655011, China; 3 College of Information Engineering, Hainan Vocational University of Science and Technology, Haikou 571126, China; 4 Department of Computer Science, COMSATS University, Islamabad 45550, Pakistan; 5 BK21 Chungbuk Information Technology Education and Research Center, Chungbuk National University, Cheongju-si 28644, Republic of Korea; 6 Department of Computer Science, Al Ain University, Abu Dhabi 64141, United Arab Emirates

**Keywords:** deep learning, medical image diagnosis, lung cancer, computed tomography (CT), computer-aided diagnostic system (CAD), gray wolf optimization (GWO), genetic algorithm (GA), transfer learning, classification, segmentation

## Abstract

Artificial intelligence plays an essential role in diagnosing lung cancer. Lung cancer is notoriously difficult to diagnose until it has progressed to a late stage, making it a leading cause of cancer-related mortality. Lung cancer is fatal if not treated early, making this a significant issue. Initial diagnosis of malignant nodules is often made using chest radiography (X-ray) and computed tomography (CT) scans; nevertheless, the possibility of benign nodules leads to wrong choices. In their first phases, benign and malignant nodules seem very similar. Additionally, radiologists have a hard time viewing and categorizing lung abnormalities. Lung cancer screenings performed by radiologists are often performed with the use of computer-aided diagnostic technologies. Computer scientists have presented many methods for identifying lung cancer in recent years. Low-quality images compromise the segmentation process, rendering traditional lung cancer prediction algorithms inaccurate. This article suggests a highly effective strategy for identifying and categorizing lung cancer. Noise in the pictures was reduced using a weighted filter, and the improved Gray Wolf Optimization method was performed before segmentation with watershed modification and dilation operations. We used InceptionNet-V3 to classify lung cancer into three groups, and it performed well compared to prior studies: 98.96% accuracy, 94.74% specificity, as well as 100% sensitivity.

## 1. Introduction

Numerous environmental factors, including chemical exposure, poisonous gases, smoking, as well as high alcohol intake, have been linked to the emergence of uncommon mutations in human DNA [1]. These mutations cause transcriptional changes that result in abnormal proteins, disrupting cell growth and, ultimately, the body [2]. Lesions may develop in any part of the human body, including the lungs, skin, breasts, as well as the brain. The defective cell changes that cause these lesions are called DNA mutations. Cancer is the second biggest cause of mortality worldwide, behind heart disease, as reported by the World Health Organization (WHO). Preventing and controlling cancer has risen to the forefront of medical research and practice because of its enormous financial toll on individuals, families, and communities [3]. The battle with cancer relies on three primary pillars: (1) the creation and implementation of cancer preventive measures; (2) the creation and implementation of new techniques for early detection; and (3) the creation and implementation of novel medications and treatments for the treatment of cancer. Lung cancer represents the most lethal form of the disease, accounting for about one in every three fatalities caused by cancer [4]. Common symptoms include loss of weight, sore throat, coughing, tiredness, chest inflammation, chest discomfort, and hemoptysis. The probability of experiencing various physiological symptoms in each phase is summarized in Table 1.

Scientists have been developing automated solutions to help physicians reduce their burden, improve diagnostic accuracy by limiting subjectivity, speed up analysis, and reduce medical expenditures in light of the expected increase in the number of preventative/early detection methods. Lung cancer cells can only be identified if specific characteristics are recognized and evaluated. A person’s chance of developing cancer may be estimated based on their physical appearance and other characteristics that have been noted. Nodule presence and a positive cancer diagnosis are not readily related, making this task difficult even for an experienced medical specialist. Common computer-assisted diagnostic (CAD) procedures use volume, form, subtlety, stiffness, conjecture, spherical, and other previously established features. Machine learning approaches are very effective in identifying and classifying lung cancer [6,7,8,9,10]. Conversely, they have necessitated a time-consuming manual step of feature creation, a task best left to professional radiologists with relevant domain expertise [11]. Features may be automatically extracted from the input dataset using deep learning (DL) algorithms throughout the learning process [12,13,14,15]. Convolution Neural Networks, Recurrent Neural Networks, Deep Belief Networks, and Stacked Autoencoders are only a few designs used in DL [16]. One DL technique that has shown promise for classifying lung tumors from CT scan image datasets is convolutional neural network modeling. Using pre-trained models, researchers are currently concentrating on fine-tuning DL strategies by exploiting transfer learning (TL) [17]. Many research and imaging modalities have used methods of this kind before [18].

### 1.1. Contributions

The primary goal of this research is to propose a hybrid technique that uses convolutional neural network (CNN) models, the TL approach, gray wolf optimization (GWO), and genetic algorithms to identify, classify, and recognize lung cancer (GA). What follows is a brief overview of the paper’s most important findings:

- This research proposes a unique mix of improved gray wolf optimization and Inception-V3 for classifying lung cancer. It is simple to construct, has a significant impact, is accurate to within a tiny margin, and performs well on issues involving optimization of search spaces for small and large sets of characteristics.
- The IGWO uses GA to find the best possible starting places for the GWO.
- IGWO has been used to select the ideal feature subset from the original dataset, reducing the redundant and unimportant characteristics, and putting the relevance of features first.
- To the best of our knowledge, the IGWO-IV3 methodology has not been evaluated or developed yet for the detection and categorization of diabetic retinopathy. Therefore, a unique combination approach based on IGWO-IV3 should significantly increase diagnostic accuracy.
- A key component of this system for classifying lung cancer is the consideration of several indicators. The evolutionary algorithm GA is used to evaluate the proposed feature selection approach. Results are verified using standard clinical verification methods.

### 1.2. Paper Organization

Rest of the article is structured as; related prior research and literature is provided in Section 2. Section 3 and Section 4 lay out and explain the many features of this proposed approach. The experimental findings and accompanying discussed in Section 5. The research is summarized and implications for the future are discussed in Section 6.

## 2. Related Work

Many researchers have used various manual and automatic approaches, techniques, and methods to extract characteristics for illness classification and prediction. Hybrid methods have been used by several groups. All of these applications were made to improve the early diagnosis of the specified disease.

The state-of-the-art performance of many deep-learning-based lung nodule classification algorithms has gradually increased over the last several years. The hybrid mammography classification method described by Abubacker et al. [19] combines genetic association rule mining with the associative classifier fuzzy neural networks. Gray-level co-occurrence matrix for 13 morphological characteristics, as well as second- and third-order wavelet decomposition for 34 statistical features. The proposed method’s overall classification accuracy is 95.1%, higher than CNN’s 93.7%. The authors utilized GARM to remove extra details from the photographs they were analyzing, and then they used ACFNN to classify the pictures as either normal or abnormal. In order to lessen the number of incorrect diagnoses of lung cancer, Ding et al. [20] employed 3-dimensional Faster R-CNN for nodule identification. The improved performance of R-CNN in object detection was impressive. For nodule feature learning and classification, it was combined with a profound contemporary convolutional neural network architecture and the DPN [21]. The foundation for finding tumors in digital mammograms was described by Martins et al. [22]. They employed the K-means method for mass segmentation and the GLCM for extracting texture information from the resulting picture segments. An SVM was then used to classify data based on the retrieved characteristics. The suggested technique was tested on the DDSM dataset, which contained digital images of screened mammograms, and achieved an accuracy above 85%. Group-based pulmonary nodule identification utilizing multi patches technique with a Frangi-filter was developed by Jiang Hongyang et al. [23] to improve performance. A four-channel 3-dimensional convolutional neural network was created to learn the radiologist-marked features using data from both sets of images. The findings of their CAD system revealed 80.06% sensitivity with a false positive rate of 4.7 for each scan and a 94.0% sensitivity with a false positive rate of 15.1. When evaluating PSO-SVM, as well as GA-SVM, for mammography analysis, reference [24] presented a hybrid genetic swarm optimization approach with SVM (GSOSVM). Extracting features from acquired mammograms using GLCM. The authors chose the top five optimization characteristics from each method. The findings demonstrated that GSO convergence outperformed PSO and GA, and that GSO-SVM achieved a higher classification accuracy (94%) than both PSO-SVM and GA-SVM.

Using transfer learning, Zhang et al. [25] overcame the issue of sparse sample size in lung-nodule categorization. The CT scan of the lung was pre-processed and then sent to the LeNet-5 model, determining if the scan was benign or malignant and how severe the malignancy was (serious or mild). The transfer learning (TL) model was validated using the LIDC-IDRI dataset. Researchers performed transformations on each nodule’s data to supplement the training data of malignant, as well as mild, classified pictures. With a 10-fold cross-validation testing technique, they correctly categorized 97% of benign and malignant nodules and 96.7% of malignancy grades. In [26], authors suggested an Effective Classification Model for cancer detection using SVM, as well as FCM methods. Gaussian with Gabor filters was used for the preliminary processing of the input CT picture. In order to partition the lung cancer ROI, the FCM was given feature extractions from the improved picture based on a Gray-Level Co-occurrence Matrix (GLCM). The SVM classifier was given the ROI characteristics to determine the cancer stage. Experiments conducted on the LIDC-IDRI benchmark dataset showed that the suggested model had a 93% success rate.

Moreover, the metaheuristic technique known as evolutionary computing (EC) has also garnered much interest. In order to pick features, many EC-based techniques have been proposed. The genetic algorithm (GA) was used for feature selection by the authors of [27]. Binary particle swarm optimization (PSO) was suggested for feature determination by the authors in [28]. Tabu search was one method that the authors looked at for use in feature selection [29]. Gray wolf optimization (GWO) is a fresh EC approach compared to the established methods [30]. Wild gray wolf social hierarchy and hunting techniques served as the inspiration for GWO. Its better search skills are effectively used for a wide variety of real-world issues, such as those involving the optimum reactive power dispatching [31], the approximation of surface acoustic wave parameters [32], and the design of a static VAR compensator [33]. It is important to stress that the starting population of the first GWO is randomly generated. This means that wolf packs throughout the whole search region can be too similar to one another. Extensive research has revealed that the global convergence rate and the intended output for flock intelligence optimization approaches are highly influenced by the performance of the starting population. Because of this, optimization strategies focusing on the starting population have a better chance of success when the starting population is large and diverse. Given the promise of this fundamental idea, we set out to use GA to breed a more substantial foundational group. The heterogeneous population was then used in conjunction with a binary version of GWO for performing feature selection.

In contrast, it has been suggested that the IGWO strategy be used in tandem with Inception-V3 to further improve the system’s classification efficiency. In order to maximize the IV3 classifier’s capacity for accurate classification, the suggested IGWO-IV3 method would evaluate several feature combinations. The IV3 classifier will be trained by IGWO using the best possible collection of features.

## 3. Methods

**Figure 1:**
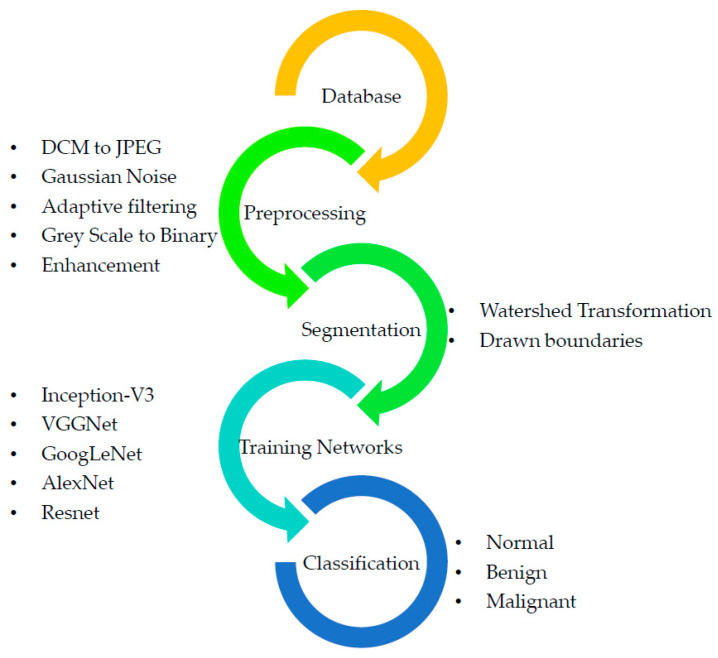
Proposed methodology.

The experiment used the LIDC-IDRI dataset [34]. The dataset, originally in ‘Dicom’, is downsized and converted to ‘jpeg’, and a new dataset is created at the intermediate level. Figure 1 presents the proposed data collection and analysis approach to data collection and analysis that has been discussed. The first phase of this technique involves retrieving a lung scan picture from a database and then processing it using adaptive filtering [34]. This lessens the impact of noise in addition to any extra aberrations that might have occurred while capturing the picture. Then, depending on the look and motion of pixels, the Feature Extraction process is carried out using the improved GWO technique. Finally, an enhanced Inception-V3 classifier categorizes tumors as normal, benign, or malignant. Performance metrics are produced to aid in a more precise interpretation of the results.

### 3.1. GWO

**Figure 2:**
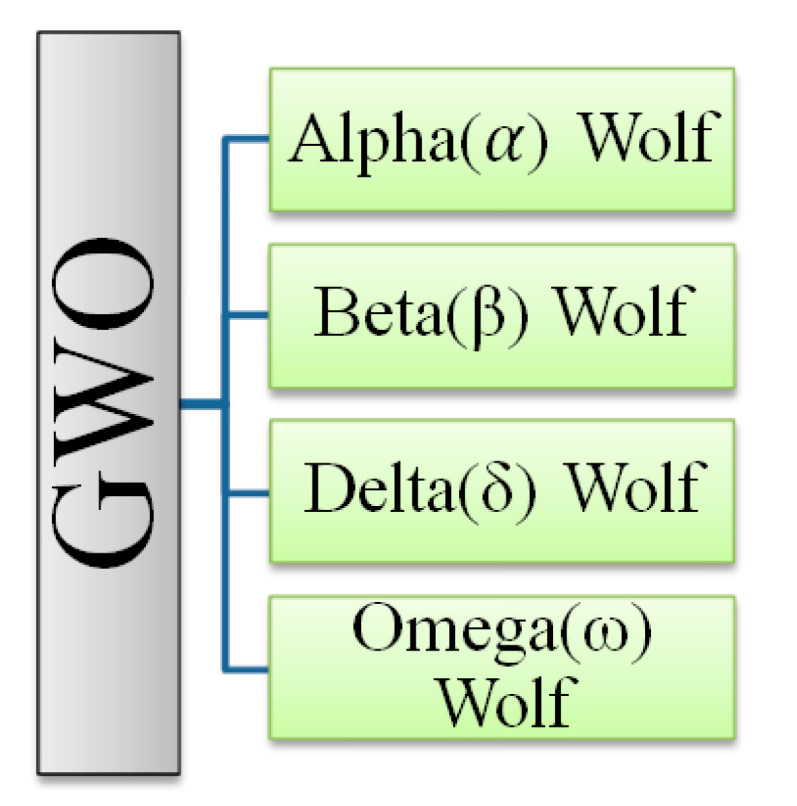
Hierarchy of the gray wolves (GW) [30].

In 2014, the Gray Wolf Optimizer (GWO) was suggested by Mirjalili et al. [30]. It is a relatively new addition to the metaheuristic algorithms that take natural cues. In this way, it resembles the hunting and pack-leading styles of gray wolves. Like all other Canidae family members, the gray wolf has a tight social order. Packs of 5–12 wolves are optimal for hunting. The traditional GWO makes certain assumptions in order to run an efficient simulation, such as the fact that there are four tiers (α, β, δ, and ω) in the wolf social hierarchy. Figure 2 shows the distinct (or hierarchical) social structure of gray wolf packs.

**Figure 3:**
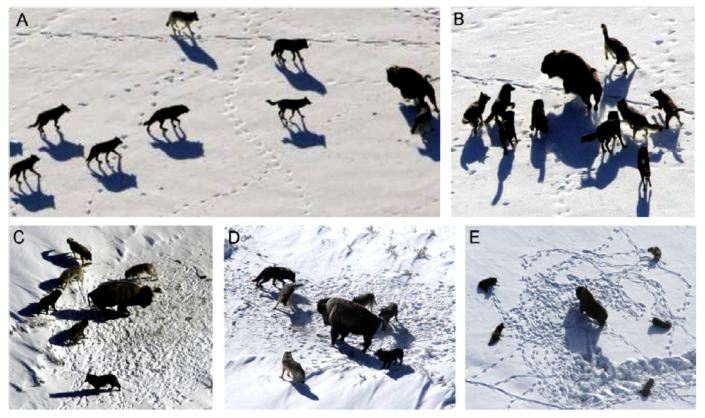
Hunting behavior of gray wolves: (**A**) chasing, approaching, and tracking prey (**B**–**D**) pursuing, harassing, and encircling (**E**) stationary situation and attack [35].

The alpha wolf is responsible for choosing where the pack will hunt, where the pack will sleep, and how the pack will behave. The pack follows the leader’s decisions without question. An exciting aspect of pack leadership is that the alpha need not be the most vital member of the pack. Second, the betas are the wolves in the pack that aid the alpha in making decisions and acting. The beta relays the alpha’s instructions to the pack and provides feedback. The gray wolf has the third-lowest ranking, omega. Omega can complete the pack and maintain the pack’s primary style. Each pack member is accountable for relaying information accurately to others. The other wolves are known as delta. Delta wolves are third in the pack hierarchy after the alpha and beta, but they are the ones that ultimately have control over the omega. Delta are responsible for keeping watch to protect the pack’s safety [30]. The gray wolf has a number of fascinating social behaviors, one of which is group hunting, which is distinct from the social hierarchy that wolves exhibit. Figure 3 depicts, from left to right, the key stages of a gray wolf hunt, as described by Muro et al. [35].

Mathematical modelling of wolf pack dynamics is the foundation for the GWO algorithm. The best answer is located at the highest level of social hierarchy, or alpha. The same holds for beta and delta, which are ranked as the second- and third-best options. After the alpha, beta, and delta wolves, omega wolves are believed to be the next possible answers [30].

#### 3.1.1. Encircling Prey Mechanism

The positions of *α*, *β*, and *δ* wolves are used to illustrate changes to the positions made by the mechanism during optimization. This is quantitatively expressed in Equations (1) and (2).

(1) $$\vec{D}=|\vec{C}\cdot {\vec{X}}_{p}\left(t\right)-\vec{X}\left(t\right)$$

(2) $$\vec{X}\left(t+1\right)={\vec{X}}_{p}\left(t\right)-\vec{A}\vec{D}$$

where *t* is the present iteration number, ${\vec{X}}_{p}$ is the location of the prey, $\vec{X}$ is the location of the wolves, $\vec{A}\;\mathrm{and}\;\vec{C}$ are the vectors representing the coefficients, and $\vec{a}$ linearly decreases from 2 to 0 as the number of iterations decreases. It is a tool for getting closer to the optimal answer. Below, in Equations (3)–(5), ${\vec{r}}_{1}$ and ${\vec{r}}_{2}$ stand for the randomized vectors between 0 and 1.

(3) $$\vec{A}=2\vec{a}.\vec{r}-a$$

(4) $$\vec{C}=2{\vec{r}}_{2}$$

(5) $$\vec{a}=2\left(1-\frac{y}{T}\right)\;$$

**Figure 4:**
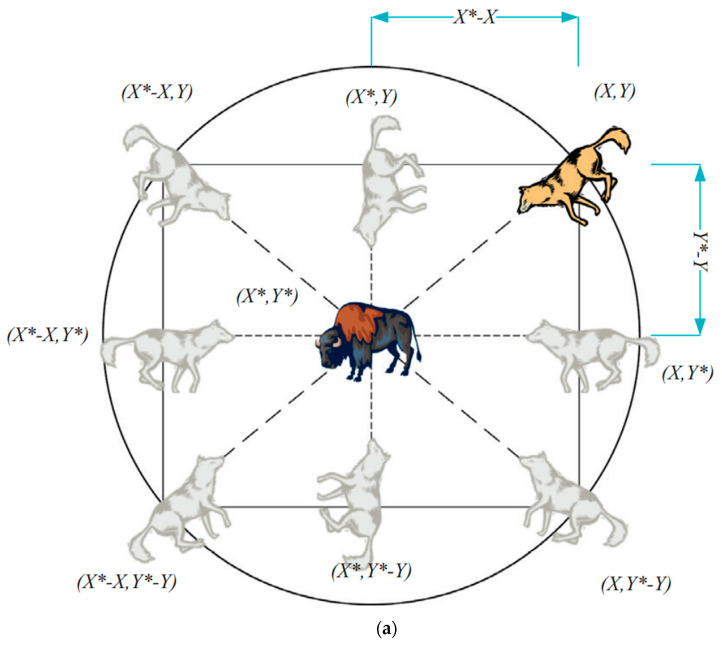

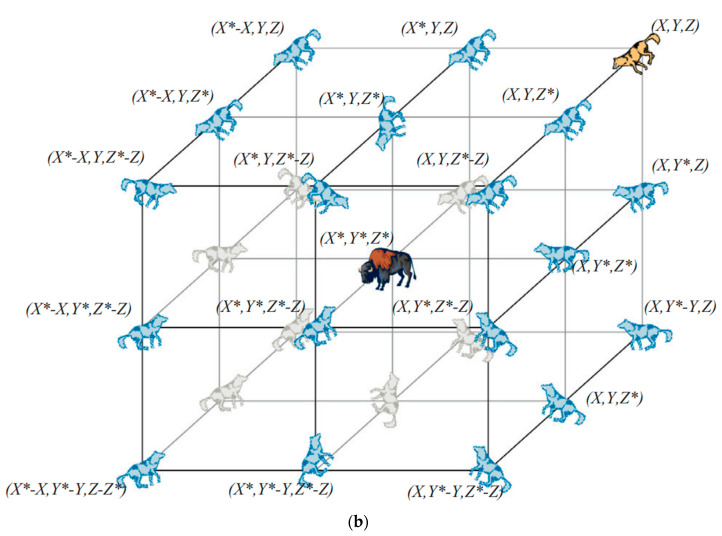
(**a**) 2D and (**b**) 3D position vectors, as well as their possible next locations [30].

Figure 4a provides an illustration of a 2D position vector, as well as some of the potential neighbors, so that the impacts of Equations (1) and (2) can be seen. From this diagram, we may infer that a gray wolf at position $\left(X,Y\right)$ can adjust its location to match that of its prey $\left(X^{*},Y^{*}\right)$. Simply changing the magnitude of the $\vec{A}$ and $\vec{C}$ vectors allow the best agent to move to a new location relative to the present one. For instance, if $\vec{A}=\left(1,0\right)$ and $\vec{C}=\left(1,1\right)$, then $\left(X^{*}-X,Y^{*}\right)$. Figure 4b shows the several probable current locations of a GW in three-dimensional space. To reiterate, wolves are free to go to any site within the range depicted in Figure 4 thanks to the random vectors *r*_1_ and *r*_2_. Using Equations (1) and (2), a GW may adjust its position inside the region surrounding its prey (2). It is possible to apply the same concept to a search space of any dimensionality, where the gray wolves roam around the current best answer in hyper-cubes.

#### 3.1.2. Hunting the Prey

Gray wolves can readily surround their prey by pinpointing its exact position. The wolf acts as a hunt master, orchestrating every step of the hunt. The gray wolf pack has a clear hierarchy of *α*, *β*, and *δ* wolves, all participating in the hunt. Consequently, the *α*, *β*, and *δ* wolves adjust their positions to where they think they should be. Equations (6)–(8) provide the mathematical expression.

(6) $${\vec{D}}_{\alpha }=\left|{\vec{C}}_{1}.{\vec{X}}_{\alpha }-\vec{X}\right|,{\vec{D}}_{\beta }=\left|{\vec{C}}_{2}.{\vec{X}}_{\beta }-\vec{X}\right|,\;{\vec{D}}_{\delta }=\left|{\vec{C}}_{3}.{\vec{X}}_{\delta }-\vec{X}\right|$$

And

(7) $${\vec{X}}_{1}={\vec{X}}_{\alpha }-{\vec{A}}_{1}.{\vec{D}}_{\alpha ,}{\vec{X}}_{2}={\vec{X}}_{\beta }-{\vec{A}}_{2}.{\vec{D}}_{\beta ,}{\vec{X}}_{3}={\vec{X}}_{\delta }-{\vec{A}}_{3}.{\vec{D}}_{\delta }$$

Gray wolves’ current location can be determined using Equation (7).

(8) $$\vec{X}\left(t+1\right)={\vec{X}}_{1}\left(t+1\right)+{\vec{X}}_{2}\left(t+1\right)+{\vec{X}}_{3}\left(t+1\right)/3$$

**Figure 5:**
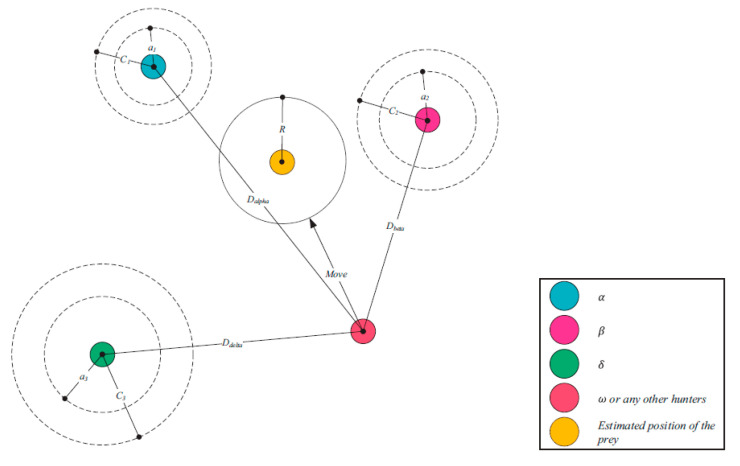
Position updating in GWO.

At this point, the position vectors of the three best solutions at the current iteration are represented by the letters ${\vec{X}}_{\alpha }$, ${\vec{X}}_{\beta }$, and ${\vec{X}}_{\delta }$, respectively. Figure 5 illustrates how a search agent changes its location in a 2D search space based on alpha, beta, and delta values. It is possible to see that the ultimate location could be anywhere at random inside of a circle, the shape of which is dictated by the placements of *α*, *β*, and δ by where it is located within the overall search space. In other words, *α*, *β*, and δ estimate as to the location of prey, while the other wolves randomly around the prey update their locations.

#### 3.1.3. Searching and Attacking the Prey

Gray wolves wait until their victim stops moving before attacking. Specifically, the Ea vector from Equation (3). Using Equation (9), we iteratively reduce a value from 2 to 0 to generate a random vector whose elements all fall within the interval [−a-a] (9).

(9) $$\vec{a}=2-\left(2\times t/Max_{iter}\right)$$

Therefore, if A 1, the wolf will be compelled to pursue the prey in an assault, and if A > 1, the wolf will veer away from the prey in pursuit of a more suitable meal. Gray wolves will look for food based on the pack’s alpha, beta, and delta positions. Exploration and exploitation are solely determined by the A and EC vector values. With the help of the random values of A, we can cause the wolf to either go closer to or farther away from its prey. To prevent becoming stuck at a local maximum, the C vector’s random values should fall between the range [0, 2]. To further complicate matters for gray wolves, one method involves randomly adding weight to the prey. If C is more than 1, then the influence of prey is emphasized, whereas if C is less than 1, then the effect of C is stochastically downplayed. In this procedure, the A and C vectors are the most important ones to adjust. When adjusted together, they might prioritize or downplay exploitative or exploratory efforts. When all conditions are met, the GWO algorithm will conclude, and the optimal alpha wolf position will be determined. Algorithm 1 is a diagrammatic description of the influence of parameters A and C on the location updates of the wolves. The GWO algorithm’s pseudo-code is presented in Algorithm 1.
**Algorithm 1.** Pseudo-code of the GWO algorithm.set the maximum number of Iterations *I*Initialize the population *X_i_* (*I* = 1, 2, 3,…, n)Initialize *α, β, δ*Calculate the fitness of the wolves (i.e PSNR value)*X_α_*, *X_β_* and *X_δ_* = 1st, 2nd and 3rd best search Agent (S.A Filter) respectively*t =* 1;**While** (*t* < *I*)  **For** each S.A    Position Updation of the current S.A as per fitness equation of PSNR  **end for**
Update *α*, *β*, *δ*Fitness calculation of all S.AUpdate *X_α_, X_β_* and *X_δ_*T = *t +* 1 **end while**
**Return***X_α_* that is Best Filter with PSNR

### 3.2. Genetic Algorithm (GA)

Holland [36] first introduced GA, an evolutionary optimization approach for optimal performance dependent on genetics and an analogy of Darwin’s natural selection process. In GA, a population consists of chromosomes, which represent potential outcomes. Each chromosome has a number of genes encoded using the binary data 0 in addition to 1 for coding purposes. For this inquiry, we used GA to determine the starting placements of the GWO. It is detailed here how the GA startup positions go through their many stages.

- Chromosomes are generated at random during the initialization process.
- A roulette system chooses which set of parental chromosomes to utilize.
- Using a single-point crossing strategy, the offspring’s chromosomes may be constructed.
- It also consistently mutates, which is a nice feature.

The first step is to “decode” the chromosomes of the population to determine where the mutations occurred.

### 3.3. Improved Gray Wolf Optimization

Since the GWO core can ensure convergence speed by ensuring adequate exploration, as well as exploitation, throughout a search, it has quickly risen in popularity over competing methods. There is no need for the dominant α-searching agent to utilize weaker β- and δ-searching agents to update their position. This is where GWO falls short. This is a major reason why the group as a whole cannot perform to its full potential. Therefore, choosing the three most important main search agents is vital in each repetition. Here, we will first apply GA to construct the starting location of GWO. Secondly, we provide a refined GWO (IGWO) approach that speeds up the leader selection process and safeguards against early convergence due to local optimum stagnation. Agents ranked 1, 2, and 3 are developed to signify a range of remedies to locate the optimal one worldwide (to encircle the prey).

Lastly, we devise the fitness-sharing concept to broaden the range of the GWO’s possible answers. The term “fitness sharing” refers to a process through which the fitness of one search agent is pooled with the fitnesses of other search agents that are vetting the same solution (or a peak). The proposed IGWO technique combines the fitness-sharing method with the GWO core to quickly locate all of the solutions to the global objective function while preventing convergence to a local solution.

### 3.4. Transfer Learning-Based Models

Transfer learning-based classification with a small medical dataset and manual training is never advisable. Transfer learning-based models are often utilized in the medical imaging classification industry to overcome these restrictions. Models such as Inception V3 [37], VGGNet [38], GoogLeNet [39], AlexNet [40], and ResNet [41] are built on transfer learning, which allows them to generalize the information they have learned about one job to another of the same kind. Regardless of the target-domain dataset, TL intends to increase the network’s performance.

## 4. Proposed Methodology

### 4.1. Proposed IGWO-Inception(V3) Approach

**Figure 6:**
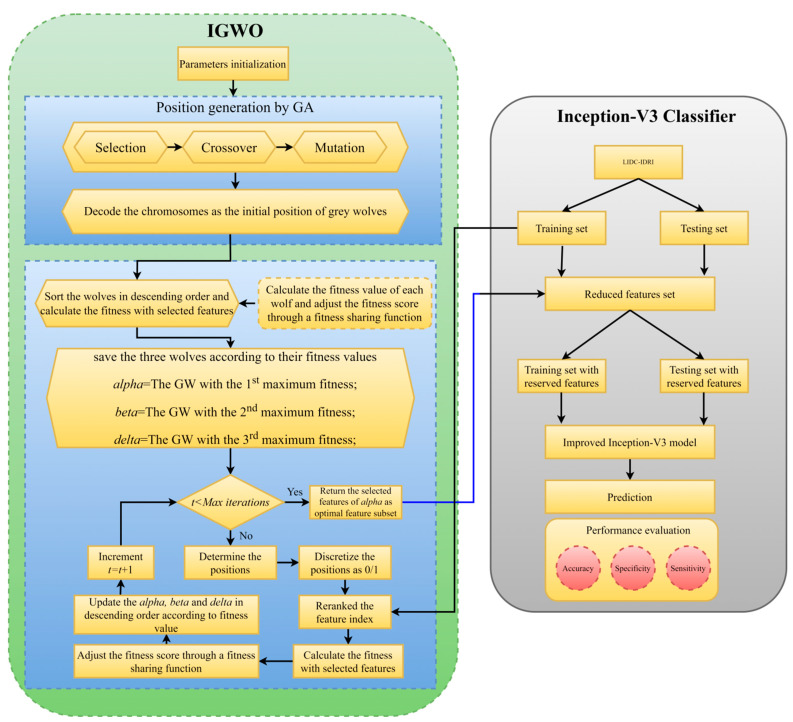
Proposed IGWO-CNN flowchart.

The categorization of lung cancer tumors using our suggested IGWO-IV3 technique is outlined in this section. The suggested solution has been divided into the following phases to make it easier to understand: (1) First, we offer the IGWO algorithm, which does not suffer from detrimental drawbacks, such as primary convergence towards the local optimum, as shown in the previous image-denoising methods. (2) The ideal collection of attributes to identify lung cancer tumors is chosen using the IGWO algorithm as a function filtering tool. (3) The IGWO approach is put out in line with transfer learning to evaluate the ideal feature combination that would maximize the transfer learning enhanced inception-V3 classifier’s classification effectiveness. Figure 6 presents the IGWO-IV3 mechanism as it was intended. The IGWO’s primary function is to dynamically probe the feature space for the best possible feature pairings. The optimal feature diversity achieves high classification accuracy with a small set of characteristics.

**Figure 7:**
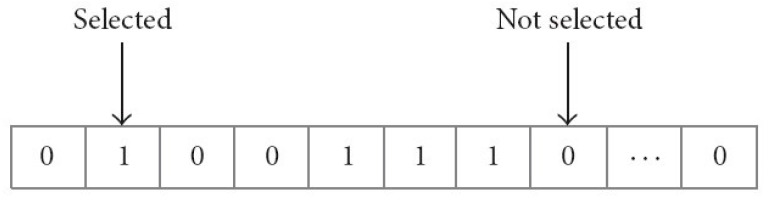
A flag vector for feature selection.

The IGWO uses the following equation for its fitness function, which is then applied to the specified characteristics to obtain an estimation of those features:

(10) $$Fitness=\alpha P=\beta \frac{N-L}{N}$$

where *P* represents the accuracy of the classification algorithm, *N* represents the total number of features present in the dataset, *L* represents the dimensions of a prioritized feature set, *α* and *β* represent two metrics of the reliability of feature selection. Both the weight and the selection feature are within the range α ∈ [0, 1] and *β* = 1 − *α*. Flag vectors for selecting features are shown in Figure 7. Real feature vectors are a subset of features represented by a standardized sequence of binary 0 s and 1 s [42]. For n-dimensional problems, the vector has n bits. Features are gathered if the bit value is equal to one, and ignored otherwise. Therefore, the total number of bits within a vector whose values are cumulatively equal to one are used to determine the size of a feature subset. Algorithm 2 displays the IGWO algorithm’s pseudocode.
**Algorithm 2.** The pseudocode of the IGWO.**Begin**
Initialize the algorithm parameters, number of iterations t, population size N, position of grey wolf p, total number of features T and mark vector of features flag.Create grey wolf’s initial positions by utilizing a genetic algorithm;Initialize α, $\;\vec{A}$, $\vec{C}$;**for**i=1:N*&* j=1:T
    **If**
$\;\;\;\;\;\;\;p\left(i,j\right)\left(\mathrm{Greater}\;\mathrm{than}\right)>\mathrm{rand}$    $\mathrm{flag}\left(j\right)=1$ **else** 0;
   **end if**

  **end for**
Estimate grey wolf fitness with selected features according to Equation (10);Estimate the fitness of each grey wolf α,β, and δ.α, β, δ are the $1^{\mathrm{st}}$$,\;2^{\mathrm{nd}}$$,\;\mathrm{and}\;3^{\mathrm{rd}}$ maximum fitness of the grey wolf recpectively;**while**k<t
  **for**
i=1:N
    Modify the grey wolf’s current position according to Equation (8);
  **end for**

  **for**
i=1:N
*&*
 j=1:T

    **If**

  $\;p\left(i,j\right)\left(\mathrm{Greater}\;\mathrm{than}\right)>\mathrm{rand}$
    $\mathrm{flag}\left(j\right)=1$ **else** 0;
   **end if**

 **end for**
(11)$$F_{i}^{\prime }=\frac{F_{i}}{m_{i}}\;\;\;i=1,\;2,\;3\ldots \ldots ,\;N\;$$(12)$$m_{i}=\sum_{j=1}^{N}share\left(d_{ij}\right)$$(13)$$Share\left(d_{ij}\right)=\left\{\begin{array}{c} 1-\left(\frac{d_{ij}}{\sigma_{s}}\right)\;\;\;\;\;\;\;\;\;\;\;\;if\;d_{ij}<\sigma_{s},\\ 0\;\;\;\;\;\;\;\;\;\;\;\;\;\;\;\;\;\;\;\;\;\;\;\;\;\;otherwse\;\;\;\; \end{array}\right.$$where $d_{ij}$ designates the Euclidean distance among the search agents i,j. And $\sigma_{s}$ represents the the radius of similarity.Compute the fitness of all search agents. Modify each search agent’s fitness using a fitness-sharing function according to Equations (11)–(13).Order the search agents according to their newly calculated fitness values in decreasing order.Estimate the grey wolves fitness with selected features according to Equation (10). Update α,β, and δ.k=k+1**end while**return the best feature subset from the selected alpha features;
  **end**


### 4.2. Classification Using Improved Inception-V3(IV3)

It is a DNN that can sort through 1000 distinct types of objects for classification. The model is first trained on a large dataset of photos, but it may later be retrained on a smaller dataset while retaining its original training data. One of the benefits of the IV3 CNN model is that it may enhance classification accuracy without requiring extensive training, significantly decreasing processing time. Layers, activation functions, and the extent to which Inception-V3 is a learnable network are all shown in Table 2. The Inception-V3 network’s principal goal is to eradicate the bottleneck demonstration between network layers, making the input size of the next layer much less. To reduce the network’s computational complexity, the factorization approach is used.

### 4.3. Performance Evaluation

Accuracy, sensitivity, and specificity are the performance evaluation indicators used to assess the quality of the suggested model. Here are their corresponding mathematical expressions:

(14) $$Accuracy=\frac{\mathrm{TP}+\mathrm{TN}}{\mathrm{TP}+\mathrm{TN}+\mathrm{FP}+\mathrm{FN}}\times 100\%$$

(15) $$Sensitivity=\frac{\mathrm{TP}}{\mathrm{TP}+\mathrm{FN}}\times 100\%\;$$

(16) $$Specificity=\frac{\mathrm{TN}}{\mathrm{TN}+\mathrm{FP}}\times 100\%$$

## 5. Experimental Results and Discussions

To test the suggested model, we used a personal computer with an E5-2609 processor, 16 GB of RAM, and a K620 Quadro graphics processing unit (GPU). Researchers used the open-source Python package Tensor flow and the Keras deep learning framework to implement the model. The suggested model’s training process utilized a Categorical cross-entropy loss function throughout 100 iterations. The k-fold cross-validation method was used to achieve classification findings that were free from bias. In this particular investigation, a 10-fold CV was used to evaluate the effectiveness of the suggested method. Despite this, conducting the 10-fold CV calculation only once will lead to an erroneous assessment. Therefore, the CV was multiplied by ten and was run ten times. Furthermore, the hyperparameters settings included a Cross Validation (K), Number of Iterations (I), Populations Size (P.S), Search domain (D), Total number of features (F), momentum (M) of 0.9, a batch size (BS) of 64, and a learning rate (LR) of 0.001, with a weight decay (WD) of 0.005. Table 3 depicts the hyperparameter setups.

### 5.1. Dataset

**Figure 8:**
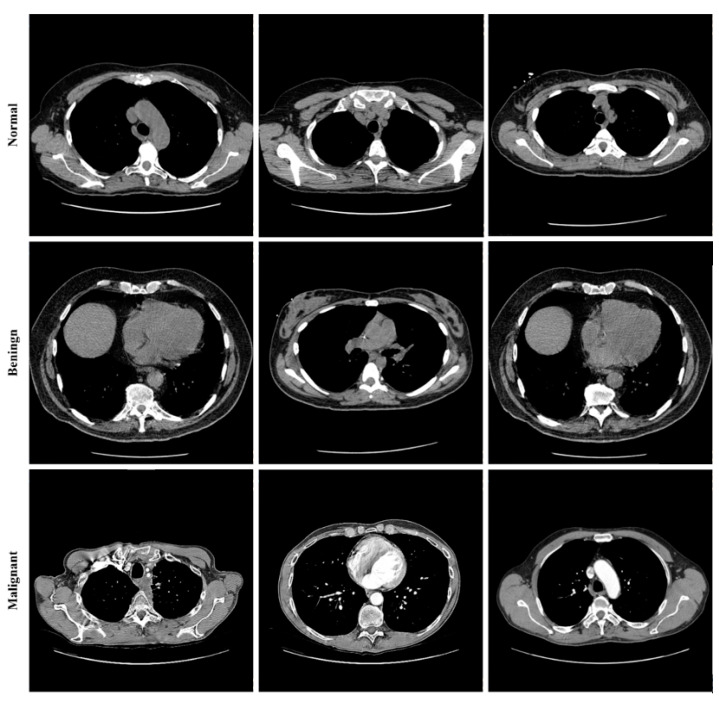
Sample images from the LIDC-IDRI dataset.

Lung cancer categorization may use a wide variety of currently accessible datasets. Lung Image Database Consortium Image Collection (LIDC-IDRI) [34], Luna16 [43], and NDSB3 (Neural Data Standardization Board, Version 3), were some of the datasets available [44]. It is possible to acquire images, analyze them, segment them, extract features from them, and classify them all with the help of a CAD system. For a CAD system to be constructed, it is necessary first to pre-process the datasets used in the project. This study used data from the LIDC-IDRI [34]. There were 910 photos utilized; 250, 320, 320 as normal, benign, and malignant, respectively. The RGB pictures were 512 pixels by 512 pixels in size. Lung cancer screening and diagnosing thoracic CT images with identified lesions made up the LIDC-IDRI. This dataset was compiled by the combined efforts of eight medical imaging firms and seven academic institutions. The data collection was initially stored in the format for Digital Imaging and Communications in Medicine (DICOM). An intermediate dataset was created by down sampling the original data and saving it in “jpeg” format. Denoising the dataset as part of the pre-processing step allowed us to obtain even better results. Figure 8 shows some images from the dataset.

### 5.2. Results and Analysis

**Figure 9:**
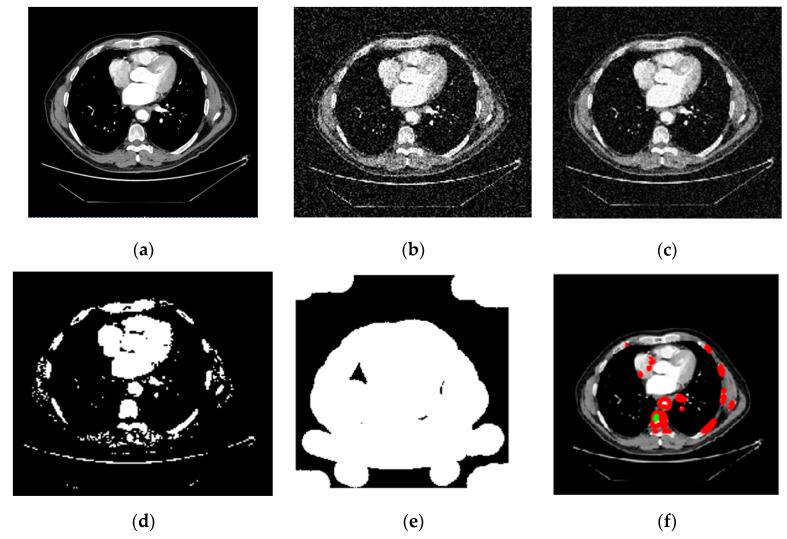
CT Image classification procedure. (**a**) Original Image. (**b**) Noisy Image. (**c**) Adaptive Filtering PSNR. (**d**) Dilate image. (**e**) Watershed Transform. (**f**) Lung cancer type: malignant.

The CT image of lung cancer used as the input, as shown in Figure 9a, is taken from the LIDC-IDRI dataset. The median filter was applied to the input picture as a first step to eliminate any artifacts that may have been produced during the image-capturing process for experimenting. It was necessary to divide the Region of Interest (ROI) into many parts in order to determine the specific area or region of interest that was wanted. In order to obtain the ROI, we first had to transform the grayscale picture into a binary form. Then, we used a morphological operation in the form of dilation, which gave us a decent but distorted image full of holes. After applying the watershed transformation or image dilation, we obtained a segmented image. This allowed us to fill in the gaps. Finally, we went back over the photos and traced the edges of the nodules.

**Figure 10:**
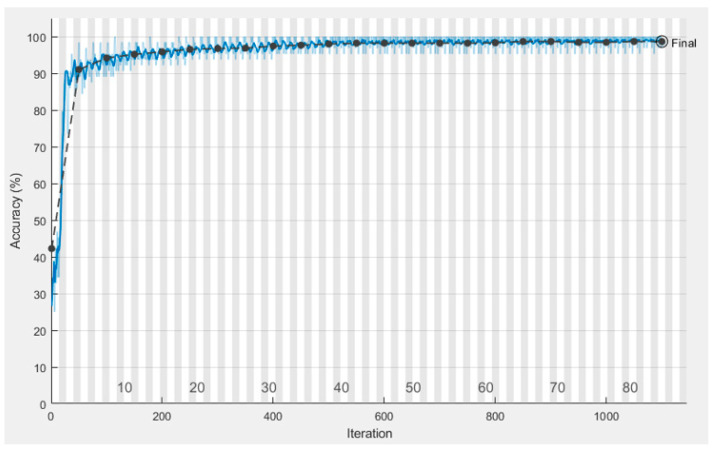
Validation accuracy.

**Figure 11:**
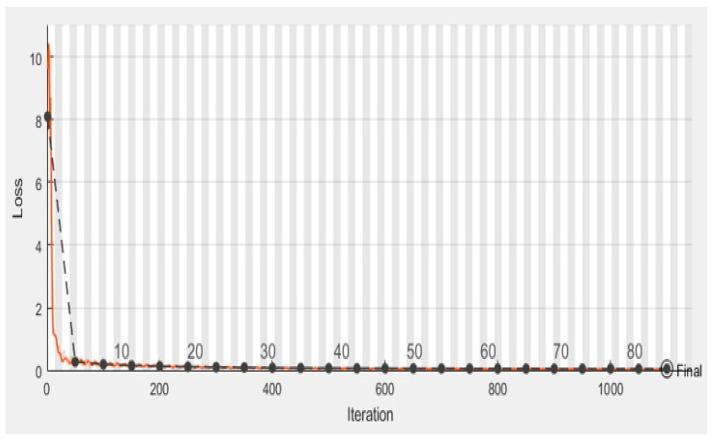
Validation loss.

We employed classification strategies in the form of Inception-V3 by using segmented pictures from each of the three classes. Figure 9a–f depicts the results of the Ct image processing with noise addition, noise reduction, dilation, watershed transformation, image segmentation, and the corresponding classification output. We began by establishing certain boundaries when starting CNN’s training process. Epochs were randomly chosen to determine the number of required iterations to achieve a high categorization accuracy. After implementing various training strategies, we achieved an accuracy of 98.96% and 0.0279 mini-batch loss value. Figure 10 depicts the training duration and validation accuracy, whereas Figure 11 depicts the accuracy and validation loss. Both Figures are located on the same page. The segmentation and preprocessing steps were reliably executed. When we first started training, we noticed that the validation accuracy eventually reached 90% once the training approached the fifth epoch.

### 5.3. Classification Results

Results showed the following benefits of the suggested technique. The proportion of false positives decreased as precision increased. In the past, methods have been used that relied on a plethora of loud, useless characteristics, which may be seen as a weakness in the reliability of the final product. In contrast to earlier research, which only included two, we included three. The accuracy of the suggested approach was 98.96%. Our three types of test data were successfully predicted using this trained TL model. The results of the prediction for varying PSNR levels across the three classes are shown in Table 4, the outcomes for each class, while our tested data included 910 scans.

### 5.4. Results Discussion

**Figure 12:**
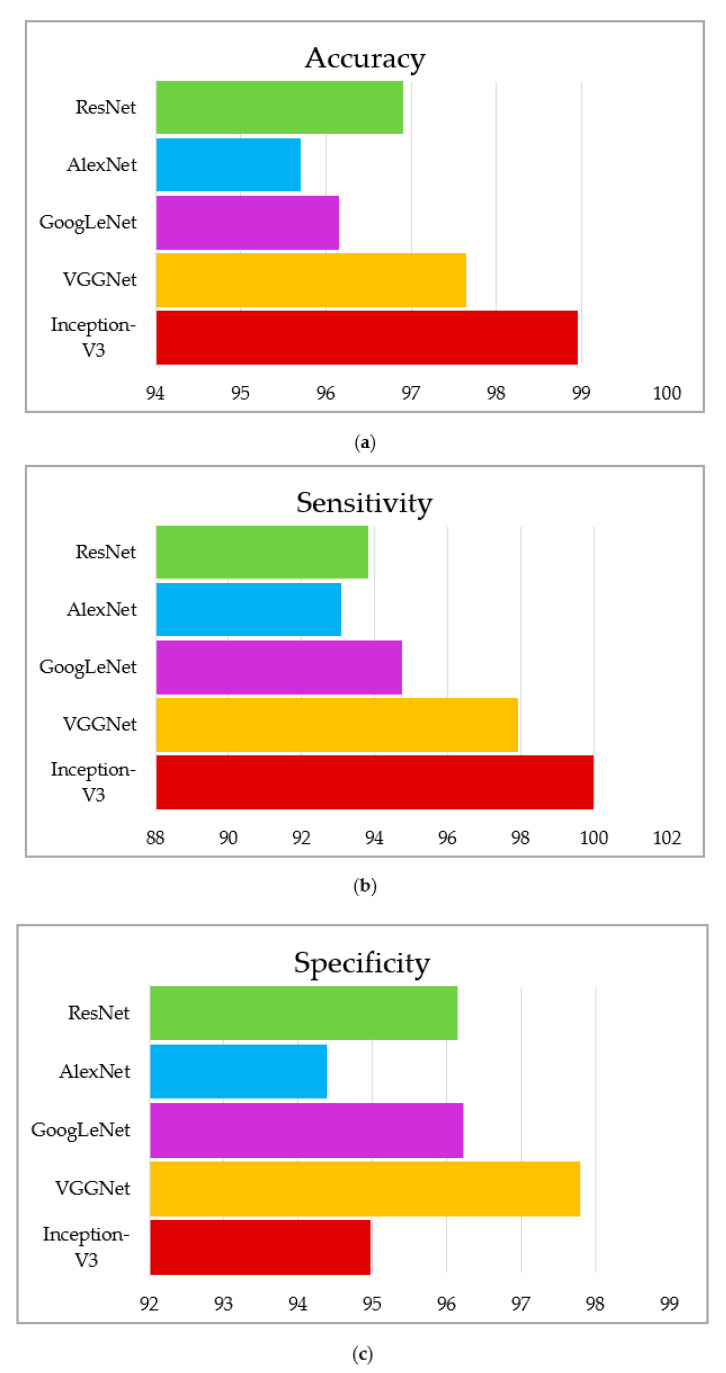
Graphical representation of IGWO-IV3, (**a**) accuracy, (**b**) sensitivity, (**c**) specificity.

**Figure 13:**
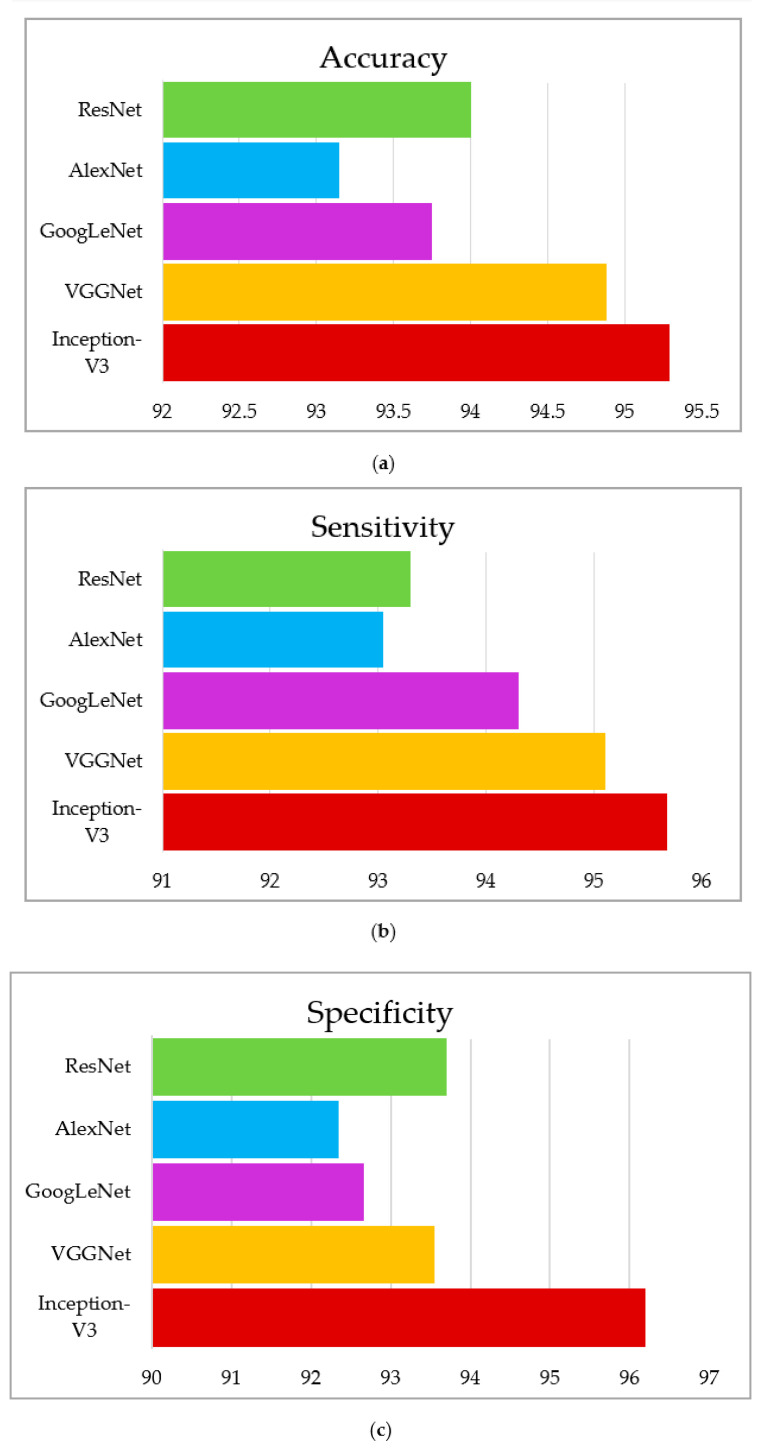
Graphical representation of GWO- IV3, (**a**) accuracy, (**b**) sensitivity, (**c**) specificity.

**Figure 14:**
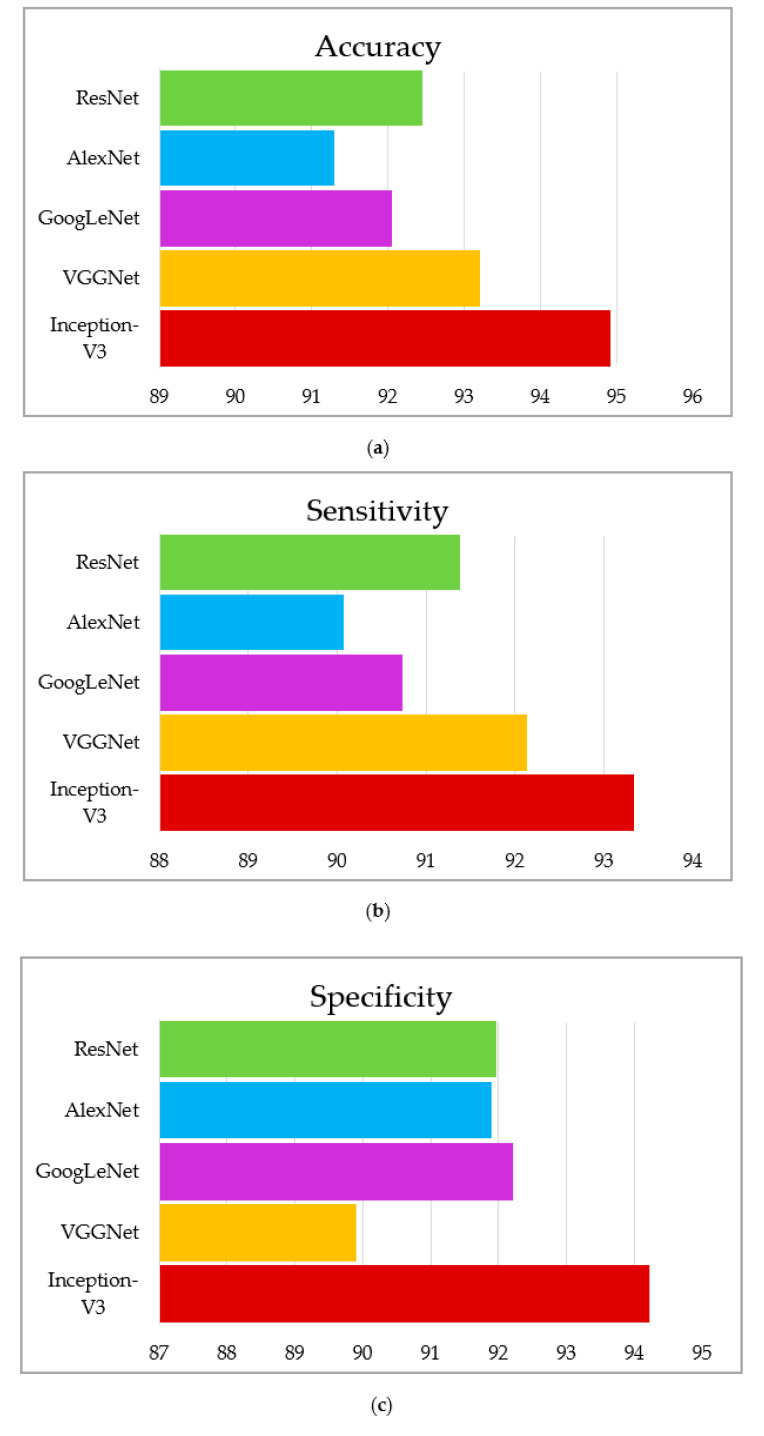
Graphical representation of GA-IV3, (**a**) accuracy, (**b**) sensitivity, (**c**) specificity.

This section summarizes the findings using the IGWO and learning-based technique developed for lung nodule identification. The proposed model is evaluated on LIDC-IDRI. The performance evaluation metrics, such as accuracy, sensitivity, and specificity, measure the proposed model’s efficacy. The proposed model’s performance is presented in Table 5, Table 6 and Table 7. It can be seen in Table 5, Table 6 and Table 7 that the IGWO-IV3 model evaluated by the LIDC-IDRI dataset achieved an average accuracy of 98.96%, 95.29%, and 94.92%, respectively. Table 5 specifies the LIDC-IDRI results, where the accuracy of Inception-V3, VGGNet, GoogLeNet, AlexNet, and ResNet is 98.96%, 97.65%, 96.15%, 95.70%, and 96.90%, respectively. Moreover, Table 6 and Table 7 specify the models GWO-IV3 and GA-IV3 evaluated on the LIDC-IDRI dataset achieved an accuracy of 95.29% and 94.92, 94.88% and 93.20%, 93.75% and 92.05, 93.15% and 91.30%, and 94% and 92.45%, respectively. The graphical representation of the models is shown in Figure 12, Figure 13 and Figure 14.

### 5.5. Comparison with State-of-the-Art Models

In this section, we present the classification performance of the proposed IGWO-IV3 approach on the LIDC-IDRI dataset. Several more recently developed and state-of-the-art lung cancer prediction models are compared.

From the results, it can be seen that our proposed methodology has the following advantages:

- Improved accuracy and reduced false positive rate.
- The previously adopted methodologies were based upon many noisy, unusable features that may have compromised classification.
- We included three classes, whereas most previous studies have only included two.

**Figure 15:**
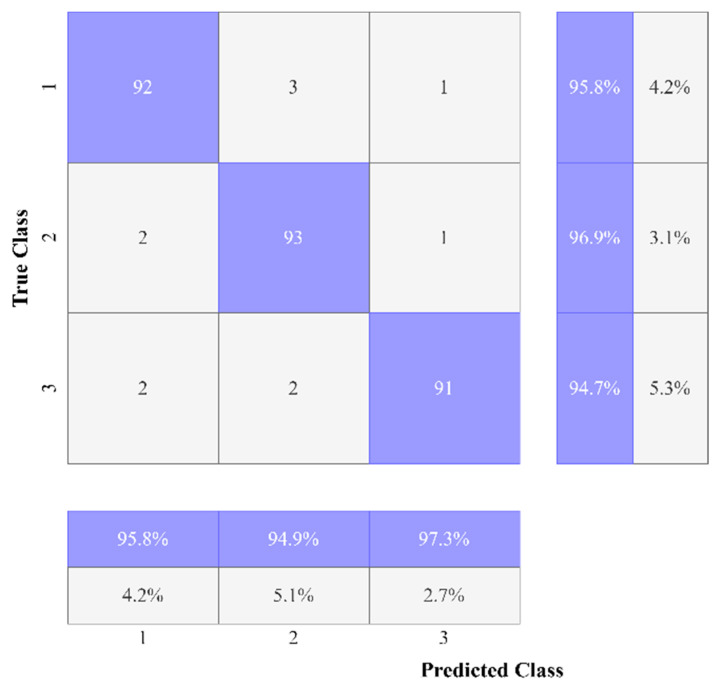
Confusion matrix of IGWO-IV3.

The proposed methodology gives the following results (Figure 15) regarding predictions:

Experiments were run between IGWO-IV3 and two other methods, GWO-IV3 and GA-IV3, to evaluate the effectiveness of the proposed method in terms of lung nodule detection. The 10-fold CV was used to determine the classification precisions of each approach, and the average values from this distribution were used in the final analysis.

**Figure 16:**
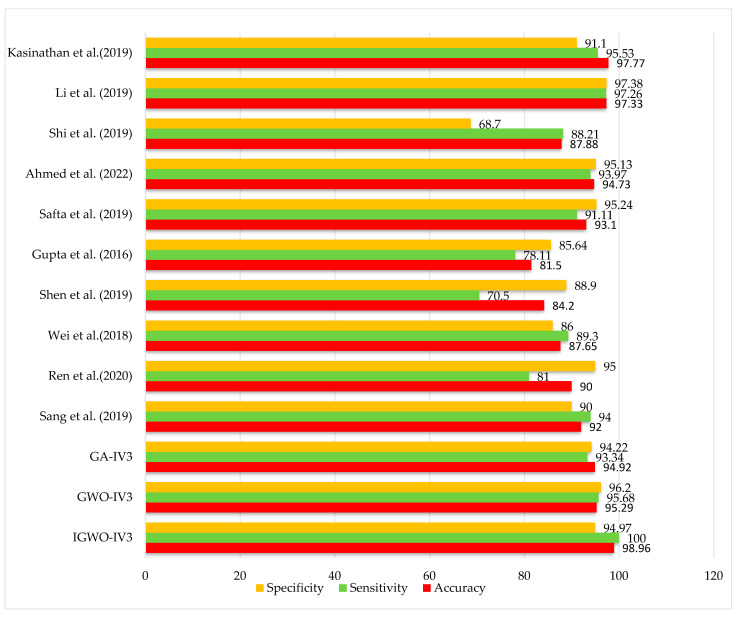
State-of-the-art comparison.

Table 8 and Figure 16 compare the planned study to primary research methods, providing examples from several databases to illustrate their findings. There is a trade-off between accuracy, specificity, and sensitivity in the measurements used for this comparison, since some studies emphasize precision while others place more emphasis on sensitivity. It was a proposed work that enhanced specificity and accuracy. However, very few studies have been sensitive enough to match the suggested study.

## 6. Conclusions

This work offers a novel IGWO-IV3 approach that uses boundary tracing to decrease false positives and improve accuracy. This work may aid detection of lung cancer by medical professionals. Our technique has the potential to be employed in a variety of settings, including the analysis of chest X-rays, the improvement of the PSNR value of fuzzy pictures captured during motion, and the categorization of other medical images. Hybridization of TL with different algorithms may or may not have produced surprising results for this dataset. This study paves the way for IGWO-IV3-based automated lung cancer detection and categorization. Features selection and categorization are the two key components of the proposed technique. First, the IGWO was used to focus in on the most critical aspects of the data. The second phase used the IV3 classifier to foresee the first-step representative feature subset. The proposed method on the LIDC-IDRI dataset was linked to well-known methodologies like GA and GWO for choosing the features, each of which employs criteria for evaluating crucial facets of the model. The results of the simulations revealed that the proposed IGWO approach achieved a quicker convergence, a higher quality solution, and a smaller feature set while still producing adequate classification results. The results demonstrated the superiority of the suggested approach at a lower computational cost.

## Figures and Tables

**Table 1:** Symptoms manifest at various stages of lung cancer [5].

Physiological Symptoms	Stage 1 (%)	Stage 2 (%)	Stage 3 (%)	Stage 4 (%)
Blood Pressure	26–43	64–87	88–91	92–94
Body temperature	54–65	33–79	92–98	94–99
Pain	26–43	30–63	34–76	43–82
Breathlessness	5–55	42–86	88–94	95–100
Anxiety	34–47	47–65	65–76	77–95
Fatigue	16–39	27–48	66–78	78–89
Irregular heart rate	13–63	18–74	75–96	97–98
Body weight loss	35–64	45–60	90–93	93–98
Constipation	11–20	19–25	27–73	43–60
Insomnia	35–47	48–62	63–87	88–92
Depression	18–31	21–46	36–78	48–83
Anorexia	-	0	36–68	66–78

**Table 2:** The Inception-V3 network’s architecture is broken down into its constituent layers: the convolutional layer (C.V), the pooling layer (P.L), the inception module (I.M), and the predictions layer (Pr. L).

Layers	Type	Activations	Learnable		
			Filter Size	Stride	Pooling Type
0	Input data	(299 × 299 × 3)	-	-	-
1	C.L	(149 × 149 × 32)	(3 × 3)	2	32
2	C.L			1	32
3	C.L	(147 × 147 × 64)		1	64
4	P.L	(73 × 73 × 64)		2	Max Pool
5	C.L	(73 × 73 × 192)	(1 × 1)	1	80
6	C.L	(71 × 71 × 192)	(3 × 3)	1	192
7	P.L	(35 × 35 × 192)		2	Max Pool
8	I.M (3A)	(35 × 35 × 256)	-	-	-
9	I.M (3B)	(35 × 35 × 288)			
10	I.M (3C)				
11	I.M (4A)	(17 × 17 × 768)			
12	I.M (5A)				
13	I.M (5B)				
14	I.M (5C)				
15	I.M (5D)				
16	I.M (6A)	(8 × 8 × 2048)			
17	I.M (7A)				
18	I.M (7B)				
19	P.L	(1 × 1 × 2018)	(8 × 8)	8	Avg Pool
20	Pr.L	(1 × 1 × 1000)	-	-	-

**Table 3:** The Details of Hyper Parameters Configurations.

Parameters		Configuration Value
K		10
I		100
P.S		8
F		N
D		[0, 1]
GA Probability	Crossover	0.8
	Mutation	0.01
Fitness Function	α	0.99
	β	0.01
M		0.90
LF		Categorical Cross-Entropy
Dropout		0.5
CW		[−1, 1]

**Table 4:** Predicted Results with PSNR values.

Number	Real Class	PSNR Value	Running Time (Sec)	Predicted Class
1	Benign	36.3867	11.2031	Benign
2		35.6983	9.8159	
3		37.5098	9.8871	
4		36.4594	10.4942	
5		36.1506	10.5559	
6		36.6885	10.0581	Normal
7		36.3145	10.3443	Benign
8		33.5808	11.1109	
9		37.8088	10.3813	Malignant
10		33.3931	11.844	Benign
11	Malignant	30.9873	11.6739	Malignant
12		33.4281	10.74	
13		36.2933	9.514	
14		33.4307	10.9254	Normal
15		36.1939	9.4905	Malignant
16		33.323	11.6049	
17		35.8181	10.6805	
18		35.113	11.5834	Normal
19		33.3303	10.0966	Malignant
20		34.7994	11.3893	Benign
21	Normal	36.13	10.7034	Normal
22		38.725	9.5804	
23		32.588	11.3979	
24		35.7119	10.6721	
25		34.9542	10.1702	
26		35.7102	10.8228	
27		35.447	11.6075	
28		38.3151	11.0796	
29		33.9639	10.85	
30		35.7997	10.3842	

**Table 5:** Performance of the proposed IGWO-IV3.

Model	Accuracy	Sensitivity	Specificity
Inception-V3	98.96	100	94.97
VGGNet	97.65	97.94	97.8
GoogLeNet	96.15	94.75	96.22
AlexNet	95.70	93.08	94.39
ResNet	96.9	93.81	96.15

**Table 6:** Performance of the proposed GWO-IV3.

Model	Accuracy	Sensitivity	Specificity
Inception-V3	95.29	95.68	96.20
VGGNet	94.88	95.11	93.55
GoogLeNet	93.75	94.3	92.66
AlexNet	93.15	93.05	92.34
ResNet	94	93.3	93.7

**Table 7:** Performance of the proposed GA-IV3.

Model	Accuracy	Sensitivity	Specificity
Inception-V3	94.92	93.34	94.22
VGGNet	93.20	92.13	89.9
GoogLeNet	92.05	90.74	92.22
AlexNet	91.3	90.08	91.9
ResNet	92.45	91.38	91.96

**Table 8:** A comparison between the proposed work and state-of-the-art models.

Author	Accuracy	Sensitivity	Specificity
Kasinathan et al. [45]	97.77	95.53	91.1
Li et al. [46]	97.33	97.26	97.38
Shi et al. [47]	87.88	88.21	68.70
Ahmed et al. [48]	94.73	93.97	95.13
Safta et al. [49]	93.10	91.11	95.24
Gupta et al. [50]	81.50	78.11	85.64
Shen et al. [51]	84.20	70.50	88.90
Wei et al. [52]	87.65	89.30	86.00
Ren et al. [53]	90.00	81.00	95.00
Sang et al. [54]	92.00	94.00	90.00
IGWO-IV3	98.96	100	94.97
GWO-IV3	95.29	95.68	96.20
GA-IV3	94.92	93.34	94.22

## Data Availability

The data supporting this study’s findings are available from the corresponding author or Anas Bilal (a.bilal19@yahoo.com) upon reasonable request.
